# Unternehmerisches Umdenken

**DOI:** 10.1365/s35764-020-00304-9

**Published:** 2020-11-09

**Authors:** Jan Recker, Frederik von Briel

**Affiliations:** 1 Universität zu Köln, Köln, Deutschland; 2 University of Queensland, Brisbane, Australien

Die Covid-19-Pandemie bringt viele Veränderungen. Es lohnt sich, bereits jetzt umzudenken für die Zeit danach.^1^

Die Covid-19-Pandemie zeigt deutlich, wie stark Umweltbedingungen sich auf den Erfolg von Gründer- und Unternehmertum auswirken. Es wird Jahre dauern, alle destruktiven Implikationen aufzuarbeiten, die die Pandemie in Volkswirtschaften und Unternehmen anrichtet.

Umweltveränderungen, inklusive der Covid-19-Pandemie, schaffen aber immer auch neue Geschäftsmöglichkeiten. Wussten Sie, dass die Hälfte der Unternehmen auf der Fortune-500-Liste 2019 während eines wirtschaftlichen Abschwungs gegründet wurde? Oder dass viele der sogenannten „Unicorns“, z. B. Airbnb, Slack und Uber, nach der globalen Finanzkrise von 2008 entstanden?

Alle Umweltveränderungen bringen die Wirtschaft aus dem Gleichgewicht, weil sie neue Nachfrage schaffen und Ressourcen von ihrer bisherigen Verwendung befreien. Dies gilt für Pandemien als auch für technologische Durchbrüche sowie regulatorischen, soziokulturellen, makroökonomischen oder politischen Wandel.

Zu wissen, *welche* Veränderungen stattfinden, *wie* sie sich auf Unternehmen auswirken und *was* zu tun ist, ist für Unternehmen von entscheidender Bedeutung, um Veränderungen nicht nur zu überleben, sondern sie auch in Vorteile umwandeln zu können. Die meisten Unternehmen implementieren in der aktuellen Krise Problemumgehungen, um danach ihren normalen Betrieb wieder aufzunehmen. Einige erkennen jedoch neue Chancen in der Veränderung und handeln entsprechend.

## Von Veränderungen kann man profitieren

Es gibt viele Beispiele für Unternehmen, die durch Umweltveränderungen entstanden sind oder davon profitiert haben. Die Abstinenzbewegung des frühen 20. Jahrhunderts führte zum Niedergang von Brauereien und Brennereien, aber auch zu einem Boom bei Erfrischungsgetränken. Coca Cola, Dr. Pepper, Pepsi-Cola und Moxie stellten sich als Nutznießer des sozialen Drucks und des gesetzlichen Verbots gegen Alkohol heraus. Sie erkannten, dass Menschen alternative Getränke suchten, als Nahrungsergänzungsmittel, um Kontakte zu knüpfen oder zur Entspannung und Belohnung. Sie erkannten, dass sich Nachfragemuster ändern würden. Und profitierten davon, als Räumlichkeiten, Maschinen und relevantes Humankapital verfügbar wurden, als Brauereien und Brennereien schließen mussten.

Ähnliche Entwicklungen sehen wir auch jetzt. Als Covid-19 Gesichtsmasken zu einem begehrten Produkt machte, verzeichneten Premiumgesichtsmaskenanbieter, wie Vogmask, Airpop und Respro, einen enormen Anstieg der Nachfrage ohne aktives Zutun. Jedoch waren die Gesichtsmasken schnell ausverkauft und die Anbieter konnten nicht von der gestiegenen Nachfrage profitieren, da ihre Lieferketten schließen mussten. Im Gegensatz dazu erfand Respilon, ein neuer Anbieter, neue Möglichkeiten, die tägliche Herstellung von Premiumgesichtsmasken und per Onlineshop zu vertreiben. Und der Shop war jeden Tag innerhalb von Sekunden ausverkauft.

## Veränderungen erkennen und vorbereiten

Nicht alle Veränderungen sind gleich. Wenn Veränderungen anstehen, ist es daher wichtig, ihre Eigenschaften zu verstehen.

**Figure 1:**
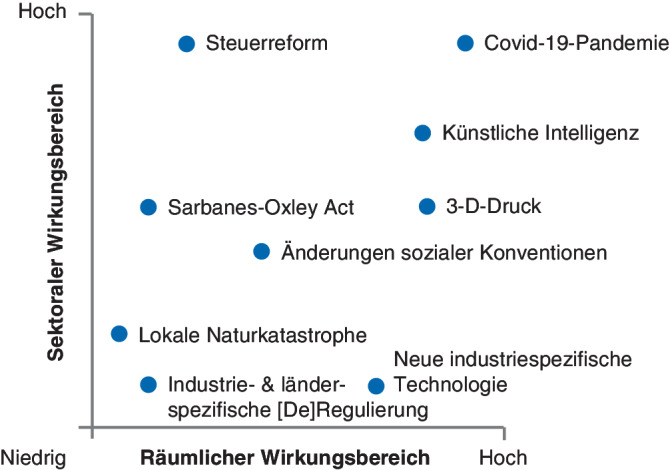

Erstens bestimmt der *Wirkungsbereich* von Umweltveränderungen ihr Änderungspotenzial. Beispielsweise betreffen lokale Naturkatastrophen, wie die australischen Buschbrände, oder branchenspezifische oder länderspezifische Vorschriften, wie der Sarbanes-Oxley Act, nur eine begrenzte Anzahl von Regionen und Branchen. Im Gegensatz dazu betreffen technologische Fortschritte, wie künstliche Intelligenz, oder globale Pandemien, die meisten Branchen weltweit. Abb. 1 zeigt Wirkungsbereichsunterschiede einiger bekannter Veränderungen.

Zweitens muss man das *Einsetzen* von Umweltveränderungen differenziert betrachten. Zum Beispiel ist eine Bevölkerungsalterung vorhersehbar, während sich der jüngste Handelskrieg zwischen den USA und China verhältnismäßig plötzlich und unvorhersehbar abspielte. Megatrends, wie der Veganismus Trend, entwickeln sich relativ langsam, während Naturkatastrophen und Virusausbrüche relativ plötzlich auftreten.

**Figure 2:**
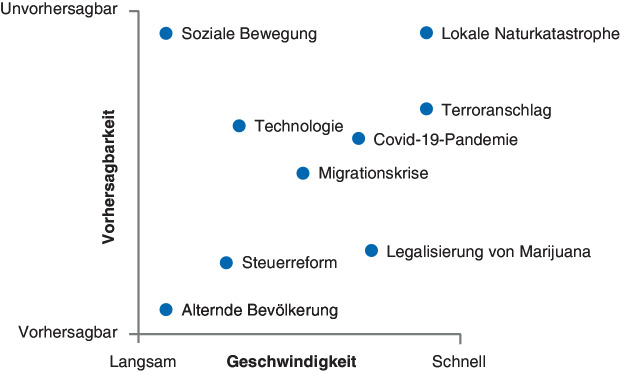

Die Covid-19-Krise zeigt, wie wichtig die differenzierte Betrachtung ist: Einige Unternehmen sahen Frühindikatoren während des langsamen Anstiegs der Virusinfektionen zu Ende 2019, passten Lagerbestände entsprechend an und bereiteten sich auf Telearbeit vor. Andere ignorierten die Frühindikatoren und wurden im Frühjahr 2020 von der globalen Welle von Infektionen überrascht. Selbst wenn Veränderungen vorhersehbar sind, kann ihre Entwicklung plötzlich erfolgen und schnelle Maßnahmen erfordern. Abb. 2 zeigt Unterschiede beim Einsetzen einiger bekannter Umweltveränderungen.

Als drittes Kriterium muss man die *Erkennbarkeit und Umsetzbarkeit* von Umweltveränderungen betrachten. Einige Auswirkungen von Veränderungen sind offensichtlich und können von fast jedem ausgenutzt werden, während andere Fachwissen oder besondere Kreativität erfordern. Zum Beispiel konnten viele Firmen relativ schnell einfache Unterstützungsdienste während der Covid-19-Krise bereitstellen, wie die Lieferung von Lebensmitteln. Aber nur wenige Firmen können das Potenzial neuer Gensequenzierungstechnologien nutzen, um vorherzusagen, wer einem Infektionsrisiko ausgesetzt sein wird. Änderungen, die knappe Fähigkeiten und Ressourcen für ihre Erkennung und/oder Nutzung erfordern, bieten mehr Potenzial für diejenigen, die auf sie einwirken können.

## Wirkungsmechanismen bewerten

Um von Veränderungen zu profitieren, muss man ihre Wirkungsmechanismen verstehen. Tab. 1 führt Mechanismen auf, die sich auf Nachfrage, Angebot, und Wertschöpfung beziehen. Wichtig ist, dass einige Mechanismen Querdenken und Gedankenexperimente erfordern. Dass Tauchmasken bei einem Mangel an medizinischen Beatmungsgeräten als Ersatz verwendet werden können, ist für die meisten erst dann offensichtlich, wenn andere darauf hingewiesen haben.

**Table 1:** 

Mechanismus	Erklärung	Beispiele
Kompression	Ermöglicht Unternehmen, Dinge schneller zu erledigen.	3‑D-Druck und digitale Prototyping-Technologien verkürzen die Entwicklungs- und Fertigungszeiten erheblich.
Konservation	Ermöglicht Unternehmen, Dinge günstiger zu erledigen.	Tools für die virtuelle Zusammenarbeit reduzieren den Bedarf an physischen Büroräumen. Online-Aufnahmen reduzieren die Zeit, die zum Wiederholen von Vorlesungen benötigt wird.
Expansion	Bietet Unternehmen mehr Ressourcen oder Nachfrage.	Die globale Finanzkrise erhöhte die Verfügbarkeit von Arbeitskräften. Die Covid-19-Krise erhöhte die Nachfrage nach Hygieneartikeln.
Substitution	Ermöglicht Unternehmen, eine Ressource durch eine andere zu ersetzen, oder lässt Kunden ein Marktangebot durch ein anderes ersetzen.	Crowdfunding ermöglicht es Unternehmen, Kredite von Kreditgebern zu erhalten, die keine Banken sind. Roboter ermöglichen es Krankenhäusern, menschliche Reinigungskräfte zu ersetzen. Pandemien führen dazu, dass Endverbraucher von normalen Kinos zu Autokinos, und von frischen zu gefrorenen Lebensmitteln wechseln.
Kombination	Ermöglicht Unternehmen die Integration anderer Marktangebote in ihre eigenen, um deren Wert zu steigern.	Lieferservices erweitern ihr Portfolio um Lebensmittellieferungen. Anbieter von Überwachungskameras fügen Funktionen zur Temperaturüberwachung hinzu. Smart-Home-Geräte stellen eine Verbindung zu Sprachassistenten von Drittanbietern her.
Generativität	Bietet Unternehmen eine Grundlage für die Erstellung völlig neuer Produkte und Dienstleistungen, Funktionen, Prozesse oder Geschäftsmodelle.	Online-Repositorien bieten Software-Quellcode, auf dem aufgebaut werden kann. Drohnen ermöglichen es, überall zu liefern. Kassenlose Geschäftstechnologie ermöglicht berührungsloses Bezahlen. Allgegenwärtige Smartphones ermöglichen On-Demand-Geschäftsmodelle.
Ungewissheits-reduktion	Reduziert die Unsicherheit von Geschäftsentscheidungen für ein Unternehmen selbst oder seine Kunden.	Blockchains verringern die Unsicherheit des Geschäftsaustauschs. Digitale Zahlungslösungen verringern die Unsicherheit von Online-Käufern.
Legitimation	Erhöht die Akzeptanz eines Unternehmens und/oder seines Angebots, sei es in Bezug auf Legalität oder Gesellschaft.	Die Legalisierung von Marihuana eröffnete neue Marktangebote. Veganismus führte zur Akzeptanz von rein pflanzlichen Lebensmitteln.
Verstetigung	Verbessert die Fähigkeit von Unternehmen, die Loyalität der Käufer und den Wert, den sie schaffen, zu erfassen.	Mit Online-Plattformen können Unternehmen über Netzwerkeffekte Mehrwerte schaffen.

## Strategisch auf Veränderungen reagieren

Wenn Sie festgestellt haben, welche Veränderungen bereits stattfinden oder stattfinden werden, und wenn Sie die möglichen Wirkungsmechanismen analysiert haben, müssen Sie Ihre Reaktion planen. Dazu müssen Sie bewerten, ob Sie durch Änderungen Ihr *Marktangebot*, Ihre *Organisation*, oder Ihre *Prozesse*, (um-)gestalten möchten.

Sie können Ihre vorhandenen *Produkte und Dienstleistungen* anpassen oder völlig neue einführen. SenseTime, ein Anbieter von Technologie für die Zugangskontrolle in Gebäuden, stellte fest, dass die Covid-19-Krise die Nachfrage nach Personen mit erhöhter Körpertemperatur erhöhte. Dementsprechend kombinierte der Anbieter Wärmebildsensoren mit vorhandenen Gesichtserkennungskameras, um diesen Anforderungen gerecht zu werden. Berücksichtigen Sie bei der Prüfung von Änderungsmöglichkeiten für Ihre Produkte/Dienstleistungen, wie sich die Bedürfnisse Ihrer bestehenden Kunden ändern, welche neuen Bedürfnisse Sie für Kunden bedienen können, und wie.

Zweitens können Sie Ihre *Organisation* anpassen. Die Covid-19-Krise hat eine beispiellose Verlagerung von Unternehmen in Richtung Telearbeit quasi über Nacht ausgelöst. Während einige Unternehmen nach Beendigung der ersten Welle wieder wie gewohnt arbeiteten, sehen andere die Gelegenheit, ihre alten Kulturen und Institutionen strategisch umzugestalten. Zum Beispiel hat Ctrip, ein Online-Reisebuchungsportal, bereits vor der Krise mit Telearbeit experimentiert. Sie stellten fest, dass ihre Callcenter-Mitarbeiter bei der Arbeit von zu Hause aus 13 % produktiver waren. Überlegen Sie sich bei der Prüfung der Änderungsmöglichkeiten für Ihr Unternehmen, welche Verhaltens- und Strukturänderungen möglich und wünschenswert sind. Welche Ihrer aktuellen Ressourcen und Kompetenzen können geändert werden, und wie? Welche neuen Ressourcen und Kompetenzen werden verfügbar?

Drittens können Sie vorhandene *Prozesse* ändern oder neue einführen. Ein Unternehmen für Bodensysteme, HTC Sweden, reduzierte die Anzahl der physischen Prototypen von fünf auf einen durch digitales Prototyping. Da Mitarbeiter aufgrund des derzeitigen Mangels an wirtschaftlicher Aktivität möglicherweise Freizeit haben, ist es eventuell ein guter Zeitpunkt, den technologischen Fortschritt zu nutzen und Ihre Prozesse durch Digitalisierung effizienter zu gestalten. Dies gilt möglicherweise auch für andere Technologien, die weitere Prozesse verbessern. Überlegen Sie, welche Ihrer Prozesse geändert, ersetzt oder beseitigt werden können und welche neuen Prozesse Sie einführen könnten, um von Änderungen zu profitieren.

## Footnotes

1 Dieser Kommentar basiert auf den Publikationen der beiden Autoren zum Thema „External Enablement“, welche in den Fachzeitschriften „Entrepreneurship Theory and Practice“ und „Academy of Management Perspectives“ veröffentlicht sind. Kopien der originalen Studien können bei den Autoren angefragt werden.

